# Histone demethylase KDM4C is a functional dependency in *JAK2*-mutated neoplasms

**DOI:** 10.1038/s41375-022-01611-3

**Published:** 2022-06-02

**Authors:** Philipp Ernst, Tina M. Schnöder, Nicolas Huber, Florian Perner, Ashok Kumar Jayavelu, Theresa Eifert, Chen-Jen Hsu, Nuria Tubío-Santamaría, Carl C. Crodel, Martin Ungelenk, Christian A. Hübner, Joachim H. Clement, Andreas Hochhaus, Florian H. Heidel

**Affiliations:** 1 Klinik für Innere Medizin 2, Hämatologie und Onkologie, Universitätsklinikum Jena, Jena, Germany; 2 Forschungsprogramm „Else Kröner-Forschungskolleg AntiAge“, Universitätsklinikum Jena, Jena, Germany; 3 Innere Medizin C, Universitätsmedizin Greifswald, Greifswald, Germany; 4 Hopp’s Kindertumorzentrum (KiTZ), Department of Pediatric Oncology, Hematology and Immunology, University Hospital Heidelberg, Heidelberg, Germany; 5 Max Planck Institute of Biochemistry, Munich, Germany; 6 Institut für Humangenetik, Universitätsklinikum Jena, Jena, Germany; 7 Leibniz Institute on Aging, Fritz-Lipmann Institute, Jena, Germany

**Keywords:** Myeloproliferative disease, Myeloproliferative disease

## Abstract

Mutations of the *JAK2* gene are frequent aberrations in the aging hematopoietic system and in myeloid neoplasms. While JAK-inhibitors efficiently reduce hyperinflammation induced by the constitutively active mutated JAK2 kinase, the malignant clone and abundance of mutated cells remains rather unaffected. Here, we sought to assess for genetic vulnerabilities of JAK2-mutated clones. We identified lysine-specific demethylase KDM4C as a selective genetic dependency that persists upon JAK-inhibitor treatment. Genetic inactivation of KDM4C in human and murine JAK2-mutated cells resulted in loss of cell competition and reduced proliferation. These findings led to reduced disease penetrance and improved survival in xenograft models of human JAK2-mutated cells. KDM4C deleted cells showed alterations in target histone residue methylation and target gene expression, resulting in induction of cellular senescence. In summary, these data establish KDM4C as a specific dependency and therapeutic target in JAK2-mutated cells that is essential for oncogenic signaling and prevents induction of senescence.

## Introduction

JAK2 is frequently mutated in the aging hematopoietic system and in myeloid cancers [1], such as myeloproliferative neoplasms (MPN). Various signaling pathways are constitutively activated by mutated JAK2-kinase. JAK-inhibitors are well tolerated and highly effective in reducing pro-inflammatory cytokine production and inflammation-related symptoms. So far, their use has been restricted to rather symptomatic approaches as meaningful reduction of disease burden is rarely seen [2]. Of note, this finding is in contrast with other small molecules, as tyrosine kinase inhibitors typically induce regression of the mutated clone. Persistence of JAK2-mutated cells under treatment with JAK-inhibitors raises questions about the role of JAK2 as a driver mutation and suggests selective dependencies that may arise from aberrant cell signaling or gene expression. Genome-wide CRISPR-Cas9-based genetic perturbation screens have identified cell type specific dependencies in various cancers in an unbiased manner [3]. Recently, efforts of the Broad Institute have created databases of large-scale functional in vitro screens, identifying genetic vulnerabilities in human cancer cell lines (DepMap; https://depmap.org/; [4]). However, validation of these targets under conditions of targeted therapies remains a necessity.

## Material and methods

### Cell lines and culture conditions

Cell lines were purchased from DSMZ (Braunschweig, Germany). Cells were cultured according to standard protocols and tested negative for mycoplasma. For proliferation assays, the number of cells was counted following trypan blue exclusion. Apoptosis was measured by flow cytometry using Annexin V/Sytox Blue staining.

### Animal studies

Mice were housed under pathogen-free conditions in the Animal Research Facility of the University Hospital Jena, Germany. All experiments were conducted after approval by the Landesverwaltungsamt Thüringen (02-030/2016). NOD-Prkdcscid-IL2rgTm1/Rj (NXG) mice were obtained from Janvier Labs (Le Genest-Saint-Isle, France). HEL cells were genetically modified and subsequently injected at equal distribution into recipient mice. Therefore, no randomization was necessary. Due to the analysis in paired samples (cells transduced with either sgRNA against KDM4C or non-targeting control (sgLuc)), no blinding was necessary. Sample size and experimental schedule were calculated assuming a relevant difference in means of survival. We used a one-sided *t*-test at *a* = 0.05 and a power of >80% with an expected difference in means of 1.75 SD (standard deviations) based on previous experience with xenotransplantation of HEL cells. Equal numbers of 8–12 weeks-old male and female mice were used for experiments in all groups.

### Primary patient samples

Primary MPN patient samples and healthy donor controls were obtained after informed consent and according to the Helsinki declaration from the Hematology Tumor Bank Jena and Greifswald, approved by the respective local ethics committees (University Hospitals Jena 4753-04/16 or Greifswald BB199/20).

### Western blot

Western Blotting was performed according to standard protocols as previously published [5, 6]. Cell lines and whole bone marrow cells were lysed as described previously. All antibodies are indicated in the Supplement.

### Quantitative PCR (Q-PCR)

RNA-preparation, reverse transcription and Q-PCR measurement were performed as described before [7, 8]. Q-PCR primers are provided in the Supplement.

### Staining and quantification of SA-ß-Galactosidase Activity

Staining of SA-ß-galactosidase (SA-ß-gal) in cells was carried out in triplicates using Histochemical Staining Kit (CS0030-1KT, Sigma-Aldrich) according to manufacturer´s instructions. Quantitative analyses were performed using an Axioskop 2 mot plus provided with a motorized stage (Zeiss, Oberkochen, Germany) and a CX 9000 digital camera (MicroBrightField Europe, Magdeburg, Germany) and the Stereo Investigator 8.1 software (MicroBrightField). Quantification was performed using the dissector method [9]. Cells of different regions of each sample were selected randomly for software supported counting.

### Confocal laserscanning microscopy

Cells were fixed and stained as described before [10]. Anti-H3K36me3 mAB (Abcam, # ab194677) was used at 1:800 dilution and a secondary AF488 conjugated anti-Rabbit mAB (ThermoFisher, #A27034) was applied 1:2000. Microscopic evaluation was performed with the Laser Scanning Microscope LSM 980 Airyscan 2 (Carl Zeiss, Jena, Germany) and ZEN 2009 software (Carl Zeiss).

### Virus production

Lentiviral particles containing the pooled sgRNA library (see below) or sgRNAs against luciferase, RPA3 and KDM4C, respectively, were generated and virus titer was assessed as described before [9].

### Genome editing by CRISPR/Cas9

Genetic editing by CRISPR/Cas9 was performed as previously described [11, 12] unless otherwise stated. Guide RNAs were designed using the Broad GPP tool (Doench, Nat Biotechnology 2014). For cloning of sgRNA sequences, the improved-scaffold-pU6-sgRNA-EF1Alpha-PURO-T2A-RFP (ipUSEPR) vector system [13], with puromycin resistance and RFP selection marker was used. Genetic inactivation by CRISPR/Cas9 was performed as published before [10]. sgRNA sequences are provided in the Supplementary Materials. For negative selection competition assays, transduced cells were mixed with non-transduced cells at 9:1 RFP^−^/RFP+ ratio for applying selection pressure. The percentage of RFP^+^ was monitored by flow cytometry.

For the genome-scale CRISPR-Cas9 screen the human CRISPR Brunello lentiviral pooled library (Addgene, #73178) cloned into the lentiGuide-Puro vector backbone (Addgene, #52963) was used. The library includes 76,441 guide RNAs targeting against 19,114 genes and 1000 control guide RNAs. Sequenced confirmed homogeneous representation with a GINI-index 0.011. Cells were treated with 200 nM ruxolitinib (Selleckchem, Lot# S137813) and DMSO in quadruplicate. Next generation sequencing was performed on an Illumina NextSeq500 platform (75 bp, single reads) aiming for a minimum of 30Mio reads per sample. Alignment and statistical analysis of the data was performed using MAGeCK (https://sourceforge.net/p/mageck/wiki/Home/) [14], version v0.5.9.3: sgRNA counts were retrieved from raw data (fastq files) via “mageck count”. Beta scores and *p* values were generated from the counts via “mageck mle” using normalization against controls (control sgRNAs) and CNV-normalization (copy number variations in HEL cells).

### RNA sequencing

HEL-Cas9 cells were transduced with Luciferase sgRNA and KDM4C sgRNA lentiviruses analogously to the proliferation assay. 48 h after transduction, selection was initiated by adding puromycin 1.5 µg/mL to each sample and the cells were expanded for another 9 days. Transduced cells were then treated with 200 nM ruxolitinib or DMSO as a control in quadruplicates for 48 h. Total RNA was isolated from 2 × 10^6^ cells using innuPREP RNA Mini Kit (Analytik Jena AG, Jena, Germany) according to the manufacturer’s instructions. Library preparation and next generation sequencing were performed by GENEWIZ GmbH (Leipzig, Germany). For quantitative analysis raw reads (fastq files, paired end, strand-specific) were trimmed with trimmomatic (v0.39) (parameters: ILLUMINACLIP:TruSeq3-PE.fa:2:30:10, SLIDINGWINDOW:4:20) [15]. Trimmed reads were aligned to the human genome (hg38) using STAR (v2.7.4a) (default parameters) [16]. Counts were generated from bam.-files using Subread featureCounts (v2.0.1) (parameters: -pB) [17]. We used DESeq2 (v1.26.0) used to generate log2 fold changes and p values from the counts [18]. Fgsea (v1.12.0) was used to perform gene set enrichment analysis, using pathways from the GSEA website (https://www.gsea-msigdb.org/gsea/index.jsp) [19, 20]. DESeq2 and fgsea were used in R (v3.6.0).

### Statistics

Comparison of two groups was performed with the two-tailed t-test for independent samples. The inter-individual variance was similar when comparing two groups. To compare the means of more than two groups, the one-way-ANOVA was used. The degree of a linear relationship between two interval-scaled groups was determined using Pearson correlation. Kaplan–Meyer curves were used for survival analysis and were plotted using GraphPad Prism version 8.00 (GraphPad software, San Diego, CA, USA) using the log-rank test (Mantel–Cox test). Statistical analysis was performed using GraphPad software (Version 8.2.0). Sample size was calculated for xenotransplantation experiments as indicated above. Global CRISPR/Cas9 screen- and RNA-sequencing were performed in quadruplicates to facilitate statistical analysis. In general, sample size of the respective experiments (*n*-value) is reported in the respective figure and/or figure legend. Data are presented as mean ± standard deviation.

## Results

### KDM4C is highly expressed and a specific dependency in JAK2-mutated cells

**Fig. 1:**
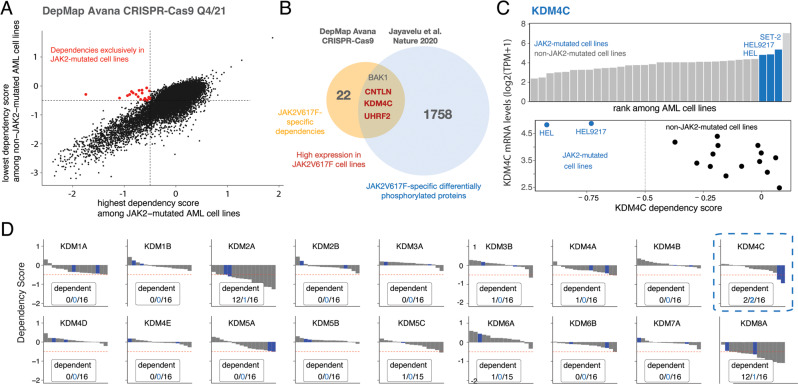
KDM4C is a specific dependency in JAK2-mutated cells. **A** Scatter plot showing the in vitro depletion scores of genes in JAK2-mutated cells versus non-JAK2-mutated cell lines according to the DepMap Avana CRISPR-Cas9 (Q4/21) database. Data points represent the median value of each sgRNA set. Scores of genes with dependencies (indicated by dependency scores < −0.5) in JAK2-mutated cells but no dependency in non-JAK2-mutated cells (dependency score > −0.5) are highlighted in red. **B** Venn diagram showing the number of JAK2-specific in vitro hits and the number of JAK2-V617F specific target genes as determined by phospho-proteome analyses [10]. Overlapping genes with high expression in JAK2-mutated cell lines are indicated in red. **C** Expression and functional dependency of KDM4C in JAK2-mutated versus non-JAK2-mutated cell lines. **D** Dependency scores of all KDM-gene-family members in myeloid cancer cell lines. Dependencies (dependency score < −0.5) in JAK2-mutated cell lines are indicated in blue, total number of dependencies in black.

Most recently, we have used in depth phospho-proteome profiling to define the global signaling landscape downstream of mutated JAK2-kinase and its perturbation by stimulation with its physiologic ligand erythropoietin or JAK-inhibitor treatment. Using RNAi-screens to explore relevant JAK2-targets, we have defined JAK2-selective vulnerabilities related to perturbation of the splicing machinery through oncogenic cell signaling [10]. To identify further vulnerabilities specifically related to mutated JAK2-kinase, we analyzed DepMap CRISPR-Cas9 datasets and identified genes that represent dependencies in JAK2-mutated hematopoietic cell lines compared with non-JAK2-mutated cells (Fig. 1A). We identified a total of 22 genes mainly related to cell signaling, apoptosis and epigenetic regulation. Of note, only 4/22 candidates had been identified as targets of mutated JAK2-kinase in phospho-proteome analysis: BAK1, CNTLN, KDM4C and UHRF2 (Fig. 1B). KDM4C was highly expressed in JAK2-mutated cell lines and was a selective dependency as compared to non-JAK2-mutated cell lines (Fig. 1C). In contrast, other members of the KDM-family could not be identified as specific vulnerabilities for JAK2-mutated cells (Fig. 1D). KDM4C (JMJD2C) is a jumonji domain-containing protein and acts as a trimethylation-specific demethylase that contributes to epigenetic regulation of both oncogene and tumor suppressor genes and is frequently overexpressed in human cancers [21, 22]. In more detail, KDM4C specifically demethylates H3K9me3/me2, H1.4K26me3, and H3K36me3/me2 via a 2-oxoglutarate-dependent dioxygenase reaction. KDM4C activity is required for development of acute myeloid leukemia [21, 22]. However, while genetic inactivation of multiple KDM4 enzymes is detrimental to normal HSCs, selective deletion of KDM4C appears to be dispensable for HSC function [23], which indicates a potential therapeutic window.

**Fig. 2:**
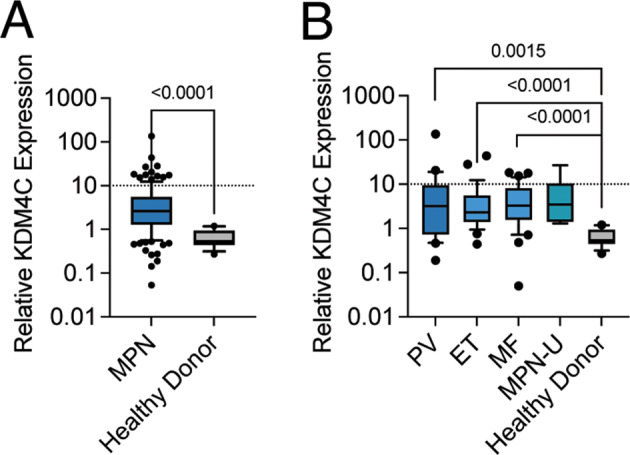
KDM4C is highly expressed in primary MPN patient cells. **A** Quantification of KDM4C mRNA expression in peripheral blood granulocytes derived from patients diagnosed with myeloproliferative neoplasms (MPN; *n* = 129) compared to healthy donor controls (*n* = 12). **B** KDM4C mRNA expression across different MPN subtypes (PV = 24, ET = 29, MF = 31, MPN-U = 6) and healthy donor controls (*n* = 12).

Regarding the high dependency of JAK2-mutated cells, we sought to investigate the functional role of KDM4C in vitro and in vivo. KDM4C was highly expressed in peripheral blood granulocytes derived from MPN patients when compared to age-matched healthy donor controls (Fig. 2A). Increased mRNA expression was detectable across all phenotypic subtypes of MPN, including polycythemia vera (PV), essential thrombocythemia (ET), myelofibrosis (MF) or unclassifiable MPN (MPN-U) (Fig. 2B).

**Fig. 3:**
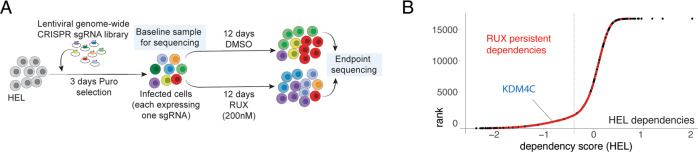
KDM4C is a JAK-inhibitor persistent functional vulnerability. **A** Schematic depicting experimental setup of the in vitro CRISPR-Cas9 screen in JAK2-mutated HEL cells. Pharmacologic treatment was performed for 12 days with 200 nM ruxolitinib (RUX) versus DMSO control. **B** Dot-plot of ranked differential CRISPR-Cas9 screening hits between the RUX-treated and control condition. The Y axis shows the gene-rank, on the X axis the differential beta scores (Δbeta-scores) are plotted. RUX-“persistent” dependencies are highlighted in red.

As indicated above, Janus kinase inhibitors are well tolerated and highly effective in reducing pro-inflammatory cytokine production and inflammation-related clinical symptoms. However, reduction of disease burden is rarely seen, which highlights the necessity to identify targets that are accessible and effective in combination with JAK-inhibitor treatment. In order to confirm the functional dependency on KDM4C under treatment conditions with the JAK-inhibitor ruxolitinib (RUX), we applied a genome-wide CRISPR-Cas9 screen in the human JAK2-mutated cell line HEL. This cell line was selected for its sensitivity to RUX and ability to be transduced among the JAK2-mutated human cell lines. For the screen, HEL cells were infected with the human CRISPR Brunello lentiviral pooled library including 76,441 guide RNAs targeting against 19,114 genes and 1000 control guide RNAs and treated with 200 nM RUX (or DMSO, as control) for 12 days (Fig. 3A). Alignment and statistical analysis of the data was performed using the MAGeCK and MAGeCK-Flute algorithms as described before [24]. Specifically, MAGeCK was used to align guide sequences from FASTQ files based on the guide-matrix. Subsequently, the MAGeCK-MLE algorithm was used to statistically compare dropout and enrichment of guides between day 0 and day 12 separately for treated (RUX) versus untreated (DMSO) conditions. Here, 749 genes were identified as functional dependencies upon ruxolitinib treatment (dependency score < −0.5). Of note, KDM4C was detected among the ruxolitinib “persistent” dependencies (HEL dependency score −0.92) (Fig. 3B). Taken together, KDM4C was identified as a selective vulnerability of JAK2-mutated cells also under exposure to JAK-inhibitor treatment.

### Inactivation of KDM4C results in impaired cellular function of JAK2-mutated cells

**Fig. 4:**
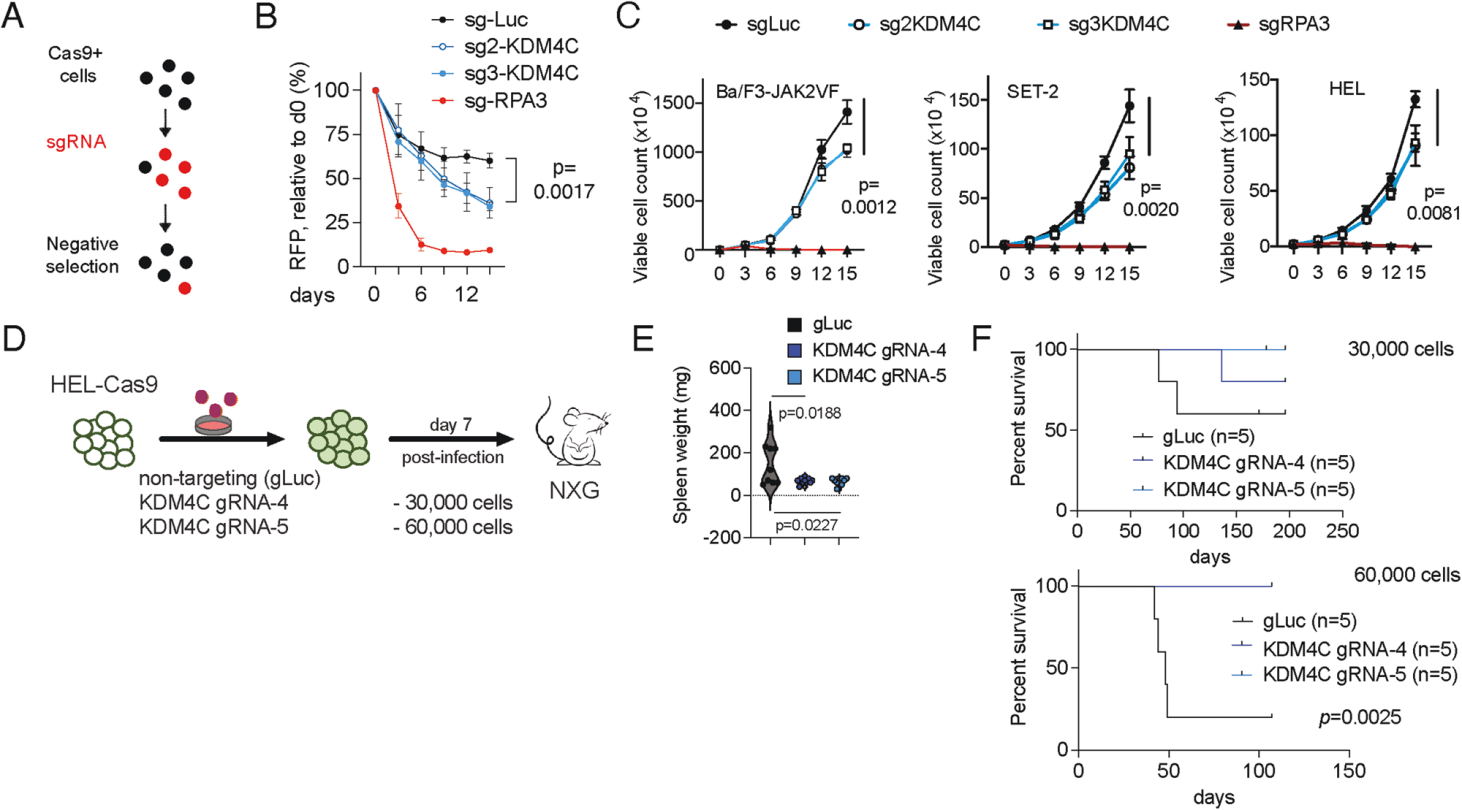
Inactivation of KDM4C results in loss of cell competition and reduced proliferation. **A** Schematic depicting negative-selection competition assay. **B** Results of the negative-selection competition assay showing the relative percentage of RFP + sgRNA+ cells over time after transduction of JAK2-mutated HEL cells with indicated sgRNAs targeting luciferase control (sg-Luc), KDM4C (sg2-KDM4C; sg3-KDM4C) or RPA3 (positive control, sg-RPA3). *n* > 3. **C** Proliferation assayed by cell counting after trypan blue exclusion for Ba/F3-V617F-Cas9-Blast cells, SET-2-Cas9-Blast cells and HEL-Cas9-Blast cells transduced with sgRNAs targeting KDM4C, RPA3, or a non-targeting control (sgLuc). *n* > 3 independent experiments. **D** Schematic representation of the experimental procedure for CRISPR-Cas9 mediated knockout of KDM4C in human HEL cells followed by transplantation into xenograft mice at two different dilutions (30,000 cells; 60,000 cells). **E** Violin plot showing spleen weight of recipient animals at the timepoint of analysis (*n* = 9; two-tailed *t*-test). **F** Kaplan–Meier survival curves of humanized NXG recipient mice injected with two different dilutions of CRISPR-Cas9 edited human HEL cells.

To further assess the functional importance of KDM4C in JAK2-mutated cells, we used a CRISPR–Cas9-mediated gene-editing and negative-selection strategy (Fig. 4A, as described before [11]). Genetic deletion was confirmed by Western blotting, and PCR. Cells expressing different KDM4C single guide RNAs (sgRNAs) were (partially) outcompeted by non-transduced cells in JAK2-mutated cell lines (HEL, *p* = 0.0017) compared to non-targeting (sg-Luc) controls (Fig. 4B). Therefore, these in vitro results suggest that KDM4C is relevant under conditions of cell competition in JAK2-mutated cell lines. To confirm these results, we aimed to investigate cell proliferation in different murine and human JAK2-mutated cells following genetic inactivation of KDM4C in vitro (Fig. 4C). Here, loss of cell competition could be attributed to impaired proliferative capacity of KDM4C deficient murine (Ba/F3-JAK2VF; *p* = 0.0012) and human (HEL, *p* = 0.002; SET-2, *p* = 0.0081) JAK2-mutated cells. To validate the functional impact of KDM4C deletion in human JAK2-mutated cells in vivo, we performed CRISPR-Cas9-mediated deletion of KDM4C in HEL cells and assessed disease dynamics after transplantation into humanized NXG mice at different dilutions (Fig. 4D). Inactivation of KDM4C reduced disease activity as indicated by reduced spleen size of recipient mice (*p* = 0.0188 and *p* = 0.0227, respectively; Fig. 4E). Moreover, deletion of KDM4C delayed disease progression at different dilutions. When injecting 30,000 transduced cells, disease penetrance was reduced in KDM4C depleted cells (gLuc: 40%; KDM4Cg4: 20%; KDM4Cg5: 0%; Fig. 4F, upper panel). At higher counts of 60,000 transduced HEL cells per animal, overall survival was significantly improved (median survival of gLuc: 48 days; sgKDM4C: not reached; *p* = 0.0025) (Fig. 4F, lower panel).

### Loss of KDM4C results in altered methylation of target histones and induction of senescence

**Fig. 5:**
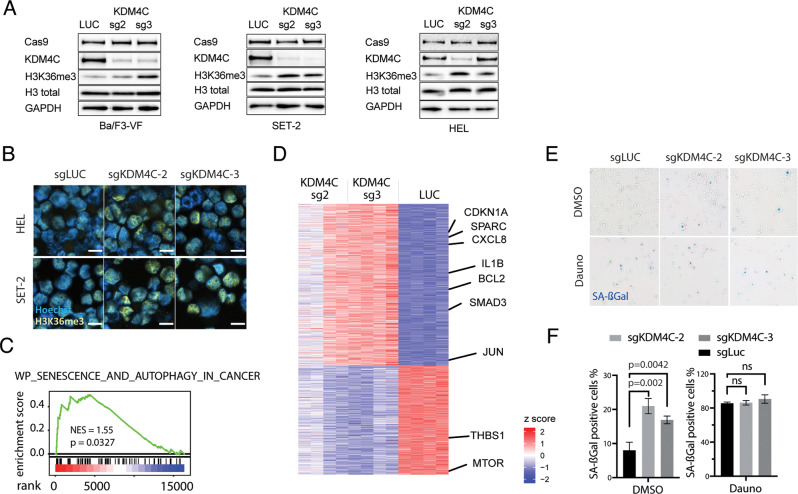
Loss of KDM4C leads to differential methylation of target histones and induction of cellular senescence. **A** Western blot analysis in BaF3/JAK2-V617F_Cas9_Blast, SET-2_Cas9_Blast and HEL_Cas9_Blast cells following CRISPR-Cas9 knockout using KDM4C specific sgRNAs (KDM4C-sg2, -sg3) or non-targeting control (LUC). **B** Immunofluorescence analysis of H3K36me3 and Hoechst-staining in SET-2_Cas9_Blast and HEL_Cas9_Blast cells following CRISPR-Cas9 knockout using KDM4C specific sgRNAs (KDM4C-sg2, -sg3) or non-targeting control (LUC). **C** GSEA showing enrichment of genes related to senescence and autophagy in cancer. Plotted are normalized enrichment scores (NES). **D** Heat map of differentially expressed genes in KDM4C deleted HEL cells versus non-targeting control (LUC); *n* = 4. Red zones represent higher gene expression (upregulation), blue zones represent lower gene expression (downregulation). **E** Representative cytospins of SA-beta-Gal-staining of KDM4C depleted HEL cells compared to non-targeting control (sgLUC). Cells exposed to daunorubicin (Dauno) serve as positive control for SA-beta-Gal staining. **F** Bar graph depicting quantification of SA-bet-Gal staining as depicted in **E**. *n* = 3 independent replicates, two-tailed *t*-test.

Genetic inactivation of the lysine-specific demethylase KDM4C resulted in increased methylation of H3K36, which is the specific residue targeted by KDM4C. This effect was detected by Western blotting (Fig. 5A) and immunofluorescence staining (Fig. 5B) in murine (BaF3-VF) and human (SET-2, HEL) JAK-mutated cells. H3K36me3 can mediate multiple transcriptional-related events, such as the regulation of transcriptional activity, is associated with both facultative and constitutive heterochromatin and has been linked to cellular processes involved in senescence [25]. When examining the transcriptome of KDM4C-depleted JAK2-mutated HEL cells, we found increased expression of CDKN1A (p21), IL1beta, BCL2, and downregulation of THSB1 or MTOR. Consistently, gene set enrichment analysis (GSEA) revealed a strong induction of gene sets related to cellular senescence (NES = 1.55; *p* = 0.0327; Fig. 5C, D). Furthermore, gene set enrichment analysis (GSEA) demonstrated enrichment of PI3K/AKT/MTOR pathway and NF-κB signaling, whereas genes encoding transcription initiation and genotoxicity pathways appeared repressed.

For functional validation, genetic deletion of KDM4C by CRISPR-Cas9 was induced and compared to daunorubicin treatment as a positive control to induce cellular senescence. Here, we found induction of H3K36me, H3K9me3 and p21 (data not shown) along with significant increase in SA-ßGal staining in JAK2-mutated HEL cells in vitro (Fig. 5E, F). These findings indicate that inactivation of KDM4C reduces cell competition and proliferative capacity and induces cellular senescence in JAK2-mutated cells.

## Discussion

In summary, we have identified lysine-specific demethylase KDM4C as a downstream effector of mutated and constitutively active JAK2 kinase. KDM4C represents a functional vulnerability in JAK2-mutated cells and its inactivation remains detrimental upon co-treatment with JAK1/2-inhibitors.

Most recently, KDM4C has been described as a regulator of stemness [26–29], cancer cell resistance [30, 31] and cancer progression [32, 33] in various cancer models and KDM4C germline variants may increase multi-cancer vulnerability through dysregulation of target histone methylation [34]. In murine models of acute myeloid leukemia (AML) driven by MLL-rearrangements, genetic inactivation of all Kdm4 family members blunted leukemia development while inactivation of Kdm4c alone showed minor effects regarding proliferation of leukemic cells [21]. In contrast, KDM4C was shown to regulate ALKBH5 expression in human AML cells by increasing chromatin accessibility to MYC and Pol II and to maintain KDM4C-ALKBH5-AXL signaling [29]. Here, inactivation of the KDM4C-ALKBH5 axis resulted in reduced proliferation, impaired colony formation and loss of leukemia stem cells. The role of KDM4-family proteins has been also investigated in detail in hematopoietic stem cells and models of acute myeloid leukemia (AML). Deletion of Kdm4 family members (Kdm4a/b/c) in murine hematopoietic stem cells resulted in accumulation of H3K9me3 on transcription start sites, transcriptional silencing and loss of stem cell self-renewal [23]. In contrast, inactivation of Kdm4c alone did not reveal deleterious effects in normal HSCs, indicating a potential therapeutic window for defining it as a target in myeloid neoplasms. Our data provides first evidence that JAK2-mutated cells may show an even greater dependency than other myeloid leukemia cell lines. Moreover, this vulnerability is uncoupled from JAK-inhibitor treatment, which is the standard-therapy for relevant subgroups of JAK2-mutated cancers. Given the fact that KDM4C was dispensable for normal hematopoietic stem- and progenitor cells, targeting this enzyme may represent a strategy to selectively target JAK2-mutated cells in myeloproliferative neoplasms.

Induction of cellular senescence has been linked to epigenetic modulator genes and specifically lysine-specific demethylases such as LSD1 and KDM4C. In melanoma cells, enforced expression of KDM4C promoted melanomagenesis through altered methylation of relevant target histone residues [35]. Targeting relevant demethylases such as LSD1 or KDM4C had deleterious effects on melanoma growth by inducing senescence. In JAK2-mutated cells, inactivation of KDM4C resulted in reduced proliferation in vitro, increased methylation of target histone residues (H3K36, H3K9) and induction of senescence. This is of major interest, as the interplay between inflammation and induction of senescence in MPN is so far not well understood. Aging of the microenvironment through MPN induced chronic inflammation may foster induction of senescence in normal, non-mutated HPSCs or niche cells such as mesenchymal stroma cells (MSC). Conversely, senescent cells may in turn contribute to the maintenance of chronic inflammation through the senescence-associated secretory phenotype (SASP). Further studies exploring the long-term effects of senescent JAK2-mutated cells in this therapeutic context are clearly needed. Finally, development of specific pharmacologic KDM4C inhibitors will allow pre-clinical validation of demethylases as relevant clinical targets in JAK2-mutated cancers.

## Supplementary information

Supplement

## Data Availability

RNA-seq data have been deposited in the Gene expression Omnibus database with accession number GSE203060. CRISPR screen data has been deposited to the Gene expression Omnibus database with the accession code GSE203059.
