# Supplementary material for: Histone demethylase KDM4C is a functional dependency in JAK2-mutated neoplasms
Source: Leukemia. 2022 Jun 2;36(7):1843–9. doi: 10.1038/s41375-022-01611-3 (PMC9252905; doi:10.1038/s41375-022-01611-3)
Supplement: Supplementary file 1 — Supplement [file 41375_2022_1611_MOESM1_ESM.docx]

**Supplementary Material**

**Histone demethylase KDM4C is a functional dependency in *JAK2*-mutated neoplasms**

Philipp Ernst ^1,2^, Tina M. Schnöder ^3^, Nicolas Huber ^3^, Florian Perner ^3^, Ashok Kumar Jayavelu ^4,5^, Theresa Eifert ^3^, Chen-Jen Hsu ^3^, Nuria Tubío-Santamaría ^3^, Carl C. Crodel ^1^, Martin Ungelenk ^6^, Christian A. Hübner ^6^, Joachim H. Clement ^1^, Andreas Hochhaus ^1^ and Florian H. Heidel ^3,7^

1 Klinik für Innere Medizin 2, Hämatologie und Onkologie, Universitätsklinikum Jena, Jena, Germany

2 Forschungsprogramm „Else Kröner-Forschungskolleg AntiAge“, Universitätsklinikum Jena, Jena, Germany

3 Innere Medizin C, Universitätsmedizin Greifswald, Greifswald, Germany

4 Hopp’s Kindertumorzentrum (KiTZ), Department of Pediatric Oncology, Hematology and Immunology, University Hospital Heidelberg, Heidelberg, Germany

5 Max Planck Institute of Biochemistry, Munich, Germany

6 Institut für Humangenetik, Universitätsklinikum Jena, Jena, Germany.

1. Leibniz Institute on Aging, Fritz-Lipmann Institute, Jena, Germany

**Q-PCR Primer sequences**

Human PCR primer sequences (5´-3´): KDM4C_for: AGCTCGATTTTCCACAGCCT; KDM4C_rev: AAACCTGGAGCTCAGCACTC; β2M_for: tgtgtctgggtttcatccatccga; β2M_rev: cacacggcaggcatactcatcttt; Murine PCR primer sequences (5´-3´): Kdm4c_for: AGCATGGAAAGCGACTTGAAA; Kdm4c_rev: TTGTGCCGGAGAAATGCAT; β-Actin_for: agagaggtatcctgaccctgaagt; β-Actin_rev: cacgcagctcattgtagaaggtgt.

**Western Blot Antibodies**

Anti-Cas9 mAB (Cell Signaling, #14697) at 1:1000, anti-H3K9me3 mAB (Cell Signaling, #13969S) at 1:1000, anti-H3K36me3 mAB (Abcam, #194677) at 1:1000, anti-Histon 3 mAB (Abcam, #8898) at 1:1000, anti-KDM4C mAB (Santa Cruz, sc-515767) at 1:1000, anti-p21 mAB (BD Biosciences, #556431) at 1:500 or anti-GAPDH mAB (Meridian Life Science, #H86504M) at 1:5000

**sgRNA sequences**

Human sgRNA sequences (5´-3´): sg2_KDM4C_for: GCAAGAGTATAATGCAACAG; sg2_KDM4C_rev: CTGTTGCATTATACTCTTGC; sg3_KDM4C_for: GGTCATCTGTGACTGAGTCG; sg3_KDM4C_rev: CGACTCAGTCACAGATGACC; sg4_KDM4C_for: TTCTCATAGCCACTAGACAA; sg4_KDM4C_rev: TTGTCTAGTGGCTATGAGAA; sg5_KDM4C_for: TCAGCTTTGGAAACAAGGAA; sg5_KDM4C_rev: TTCCTTGTTTCCAAAGCTGA; sg_LUC_for: GATTCTAAAACGGATTACCA; sg_LUC_rev; TGGTAATCCGTTTTAGAATC; sg_RPA3_for: GGTTGGAAGAGTAACCGCCA; sg_RPA3_rev: TGGCGGTTACTCTTCCAACC; Murine sgRNA sequences (5´-3´): Kdm4c_mm_gRNA2_for: caccCTGGCCGGAGGCTTACCAAG; Kdm4c_mm_gRNA2_rev: aaacCTTGGTAAGCCTCCGGCCAG; Kdm4c_mm_gRNA3_for: caccTTTGGATACCAGGATACAAG; Kdm4c_mm_gRNA3_rev: aaacCTTGTATCCTGGTATCCAAA; sg_Rpa3_for: ACGGGCCGGTCGATATACTG; sg_Rpa3_rev: CAGTATATCGACCGGCCCGT
